# Trends in Medicaid Managed Care Market Consolidation in the United States: A Cleaning Algorithm

**DOI:** 10.21203/rs.3.rs-10683656/v1

**Published:** 2026-10-12

**Authors:** Haoran Tao, Mohammed Ali, Leslie Johnson, Puneet Chehal

## Abstract

Background This study aims to develop a cleaning algorithm for the Centers for Medicare & Medicaid Services’ (CMS) Medicaid Managed Care Enrollment Report (MMCER) to improve longitudinal tracking of Medicaid managed care plans and parent firms. Methods This is a methodological study assessing approaches for matching plan names and managed care organization (MCO) parent firm names across states and over time under varying assumptions. We used annual state-level plan-specific enrollment data from the MMCER from 2016 to 2021. Analyses included Medicaid managed care markets in 11 states. Results MMCER data quality limitations complicated consistent identification of health plans and MCO parent firms over time. We developed a flexible cleaning algorithm that accounted for different levels of uncertainty when matching plan and parent firm names. Trends in plan and parent firm consolidation were generally stable across alternative matching assumptions. Conclusions This study provides a structured and reproducible approach for cleaning and standardizing MMCER data, supporting more accurate and policy-relevant analyses of Medicaid managed care market consolidation.

